# When Policy Meets Practice: The Dilemma for Guidance on Risk Assessment Under the Cartagena Protocol on Biosafety

**DOI:** 10.3389/fbioe.2019.00082

**Published:** 2019-05-01

**Authors:** Karen E. Hokanson

**Affiliations:** Department of Horticultural Science, University of Minnesota, St. Paul, MN, United States

**Keywords:** biosafety, cartagena protocol on biosafety, risk assessment guidance, AHTEG, COP-MOP, genetically modified organisms, biotechnology regulation

## Abstract

The Conference of the Parties serving as the meeting of the Parties (COP-MOP) to the Cartagena Protocol on Biosafety to the Convention on Biological Diversity decided years ago to undertake the development of guidance on risk assessment of living modified organisms (LMOs) resulting from modern biotechnology, in order to assist the Parties to the protocol to conduct risk assessments in line with the principles and methodology described therein. After many years of working through *ad hoc* technical expert groups (AHTEG) and open-ended online forum discussions, including an extensive process to test and revise the guidance document, the COP-MOP did not decide to endorse the last version of the document when it was finally presented to them. A failure to achieve consensus that the guidance, as it had evolved, is relevant and useful is seen as a potential setback for many Parties to the protocol with little to no experience with risk assessment. There are a number of reasons for the lack of success in this attempt to develop useful guidance on risk assessment, including a poorly defined and shifting purpose, misplaced expertise, and a misguided testing process, mostly perpetuated by the constraints of using processes of the Convention. These problems with the development of the Guidance on Risk Assessment of LMOs are explored here in an effort to elucidate the missteps that should be avoided and the lessons that can be learned. Most prominent is a need to rely upon the expanding past and present experiences with actual cases of risk assessments of LMOs, if there is to be any further attempt to develop guidance on risk assessment under the Convention and its protocol.

## Introduction

The Cartagena Protocol on Biosafety to the Convention on Biological Diversity is an international agreement that provides a regulatory framework for the safe handling, transport, and use of ‘*living modified organisms (LMOs) resulting from modern biotechnology that may have adverse effects on the conservation and sustainable use of biodiversity*’ (SCBD, 2000). The dual-purpose of the Cartagena Protocol on Biosafety (hereinafter referred to as “the Protocol”) as described in its introduction is to create ‘*an enabling environment for the environmentally sound application of biotechnology, making it possible to derive maximum benefit from the potential that biotechnology has to offer, while minimizing the possible risks to the environment and to human health’*. The Protocol entered into force in 2003 and since then has been ratified by 171 countries as Parties. Negotiations among the Parties over the implementation of the articles of the Protocol have since taken place during nine “Conference of the Parties serving as the meeting of the Parties (COP-MOP) to the Protocol, and negotiations will continue into the foreseeable future.

The Protocol has clearly significantly shaped the development of most national biotechnology regulatory frameworks, with impacts on a range of issues including environmental risk assessment (ERA), socio-economic considerations, and liability and redress, particularly in developing countries (McLean et al., 2012; Adenle et al., 2018). The dominating presence of the European Union (EU), which serves as a “Party” to the Protocol as does each of its 28 member states, with a decidedly first-world, highly precautionary stance on genetically modified organisms (Science for Environment Policy, 2017; Eriksson, 2018), has been particularly influential in these negotiations (Paarlberg, 2006; Adenle et al., 2018). The Protocol has serious implications for global agricultural trade and food security, making it critical that it is implemented in a practical way without perpetuating overly strict or unobtainable regulatory hurdles while effectively promoting the conservation and sustainable use of biodiversity.

**Figure 1:**
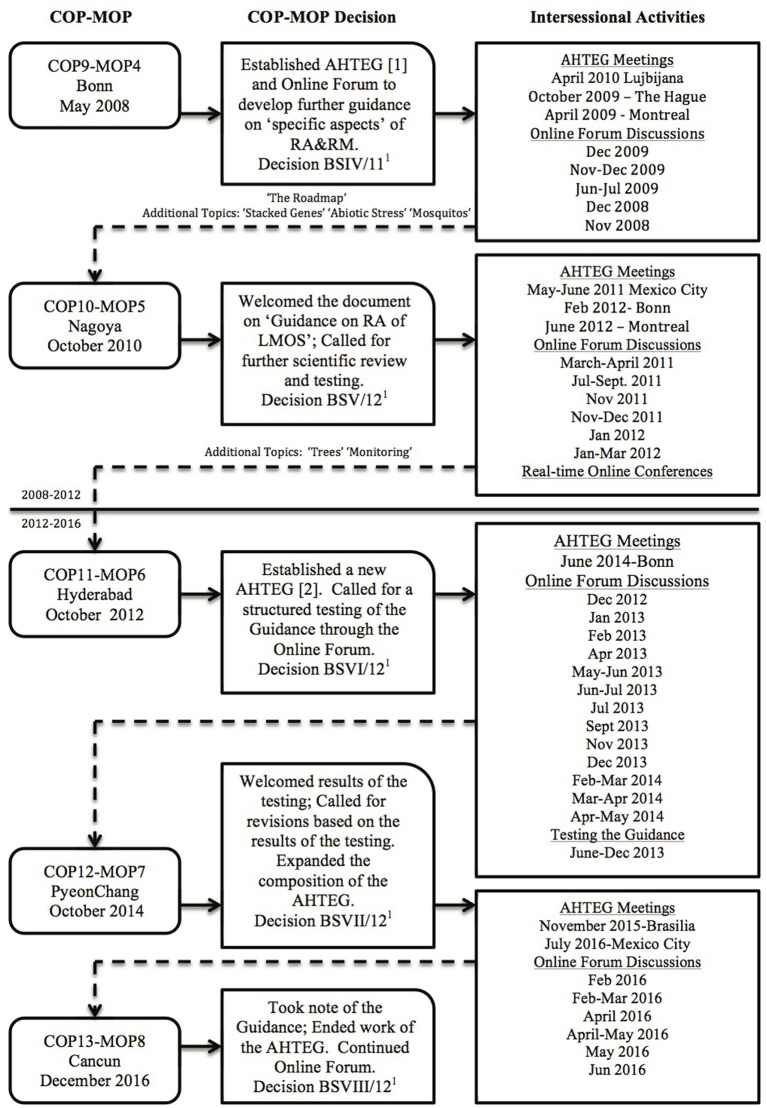
A schematic showing the timeline and activities for the development of the “Guidance on Risk Assessment of LMOs” under the Cartagena Protocol on Biosafety.

One of the most significant discussions taking place over the years of negotiation concerns risk assessment, covered in Articles 15 and 16 of the Protocol. Annex III of the Protocol outlines the objective, use, general principles, methodology, and points to consider for risk assessment (SCBD, 2000). While most Parties agree that the general principles and methodology provided in Annex III (see Box 1) represent what is typically followed for risk assessments of LMOs, some Parties saw a need to develop further guidance on “specific aspects” of risk assessment. At COP-MOP4 in 2008, the Parties agreed to establish an open-ended online forum and *ad hoc* Technical Expert Group (AHTEG) on Risk Assessment and Risk Management to develop such guidance (Decision BSIV/11)^1^ Eight years later, after various drafts of the Guidance were presented and not endorsed by the Parties over three more COP-MOPs, and frequently polarized online forum discussions and face-to-face meetings of the AHTEG, and a lengthy testing and revision process (see Figure 1), the latest draft of the Guidance on Risk Assessment of LMOs (hereinafter referred to as “the Guidance”) was completed, published and presented at COP-MOP8 in December 2016. There, the Parties decided to “take note of,” but did not “endorse” (nor “welcome,” nor “acknowledge”) the Guidance, and the AHTEG on Risk Assessment and Risk Management (hereinafter referred to as “the AHTEG”), having completed its mandate, came to a close.

Box 1 **Annex III of the Cartagena Protocol on Biosafety, Paragraphs 3-7, 8a-f.**

**Risk assessment general principles and methodology**

**General principles**

*3. Risk assessment should be carried out in a scientifically sound and transparent manner, and can take into account expert advice of, and guidelines developed by, relevant international organizations*.
*4. Lack of scientific knowledge or scientific consensus should not necessarily be interpreted as indicating a particular level of risk, an absence of risk, or an acceptable risk*.
*5. Risks associated with living modified organisms or products thereof, namely, processed materials that are of living modified organism origin, containing detectable novel combinations of replicable genetic material obtained through the use of modern biotechnology, should be considered in the context of the risks posed by the non-modified recipients or parental organisms in the likely potential receiving environment*.
*6. Risk assessment should be carried out on a case-by-case basis. The required information may vary in nature and level of detail from case to case, depending on the living modified organism concerned, its intended use and the likely potential receiving environment*.

**Methodology**

*7. The process of risk assessment may on the one hand give rise to a need for further information about specific subjects, which may be identified and requested during the assessment process, while on the other hand information on other subjects may not be relevant in some instances*.
8. To fulfill its objective, risk assessment entails, as appropriate, the following steps:
  (a) An identification of any novel genotypic and phenotypic characteristics associated with the living modified organism that may have adverse effects on biological diversity in the likely potential receiving environment, taking also into account risks to human health;
  (b) An evaluation of the likelihood of these adverse effects being realized, taking into account the level and kind of exposure of the likely potential receiving environment to the living modified organism;
  (c) An evaluation of the consequences should these adverse effects be realized;
  (d) An estimation of the overall risk posed by the living modified organism based on the evaluation of the likelihood and consequences of the identified adverse effects being realized;
  (e) A recommendation as to whether or not the risks are acceptable or manageable, including, where necessary, identification of strategies to manage these risks; and
  *(f) Where there is uncertainty regarding the level of risk, it may be addressed by requesting further information on the specific issues of concern or by implementing appropriate risk management strategies and/or monitoring the living modified organism in the receiving environment*.

The final version of the Guidance presented at COP-MOP8 includes six sections divided into three parts (Box 2), beginning with a roadmap for risk assessment (hereinafter referred to as “the Roadmap”), which is Part I. The Roadmap is meant to “elaborate on how to undertake a risk assessment” and it is the core of the Guidance (Gaugitsch, 2016). It has been the main topic of discussion during the AHTEG meetings, in the online forums, in the testing process, and at the COP-MOPs. Part II is additional guidance on specific types of LMOs (mosquitos; trees) and traits (stacked genes; abiotic stress resistance); and Part III is additional guidance on monitoring of LMOs after release into the environment. The last version of the Guidance as it was presented for discussion at COP-MOP8 can be found as an annex to the COP-MOP8 official meeting document UNEP/CBD/BS/COP-MOP/8/8/Add.1^1^, and the same document is available as Issue 4 in the Biosafety Technical Series of the Biosafety Clearing House^1^. To satisfy the concerns of some Parties, a disclaimer can also be found there:

“*Note: This publication is the outcome of the ad hoc Technical Expert Group on Risk Assessment and Risk Management at its meeting in July 2016. The views reported in this publication were not considered, discussed or otherwise approved or adopted by the Conference of the Parties serving as the meeting of the Parties to the Cartagena Protocol on Biosafety and do not necessarily represent the views of the Parties to the Cartagena Protocol on Biosafety*.”

Box 2 **The guidance on risk assessment of living modified organisms.**

**Table of contents**

Part I. 1. Roadmap for Risk Assessment of Living Modified Organisms
Part II. Specific types of LMOs
  2. Risk Assessment of Living Modified Plants with Stacked Genes or Traits
  3. Risk Assessment of Living Modified Plants with Tolerance to Abiotic Stress
  4. Risk Assessment of Living Modified Trees
  5. Risk Assessment of Living Modified Mosquitoes Species that Act as Vectors of Human and Animal Diseases
Part III 6. Monitoring of Living Modified Organisms Released Into the Environment

The document, although it was not endorsed, remains a “voluntary” guidance available for use by any Party and others who would choose to use it, although it is in no way recommended or required for a Party to follow this guidance.

In the discussions leading up to and during COP-MOP8, some Parties took the position that the Guidance was useful and practical and should be endorsed, while Parties unwilling to endorse it were critical of the process by which the document was developed, particularly because it did not allow ample opportunity for Parties to review the latest version of the Guidance before deciding to endorse or not at COP-MOP8. There were also serious concerns that the contents of the document go beyond what is consistent with Annex III of the Protocol and do not represent the years of experience gained by the Parties who have conducted actual risk assessments on LMOs. Because of this, the overall usefulness of the Guidance for the implementation of the Protocol was in question. Those Parties seemed to share an opinion that, in spite of the claims that the Guidance was not prescriptive and does not impose any obligations upon the Parties, endorsement by the COP-MOP would imply that the Guidance was recommended, if not required, for use by Parties. Because the Guidance would be more difficult to implement than more practical existing approaches to risk assessment being followed in some countries, this would likely hinder rather than enable effective risk assessment for decision-making, especially in countries with little or no experience.

This outcome was a disappointment to the Parties that called for and still perceive a need for detailed guidance on risk assessment, particularly those with little to no experience assessing the risks of LMOs currently. It is both disappointing and perplexing to all Parties to see so much effort, energy, time, and money invested into a process that failed to result in an acceptable outcome. How could this have happened? Herein are a number of observations about the process and outcome that might explain the fate of the Guidance on Risk Assessment of LMOs, and some lessons learned to help shape the process should there be attempts to develop similar guidance in the future.

## A Question of Purpose for the Guidance

The intent and purpose for the Guidance, and in particular the Roadmap, seemed to evolve significantly over the years. The topic of guidance on risk assessment was taken up early at COP-MOP2 where it was decided to establish an initial AHTEG that then met in Rome in November 2005. The Terms of Reference of the Rome AHTEG as described in Decision BSII/9^1^included to:

‘*consider the nature and scope of existing approaches to risk assessment based on national experiences and existing guidance materials;* and ‘*evaluate the relevance of existing approaches and guidance materials to risk assessment under the Protocol and identify gaps in those existing approaches and guidance materials*.

In the decision from COP-MOP2 (Decision BSII/9^1^), the COP-MOP acknowledged that:

…*‘any guidance on risk assessment and risk management developed by the Conference of the Parties serving as the meeting of the Parties to the Protocol should support a harmonized approach, in accordance with Annex III of the Protocol, taking into account internationally agreed principles and techniques developed by relevant international organizations and bodies*.’

The Rome AHTEG concluded that developing general guidance was not a priority given the amount of material already available, which needed to be collected, organized and made available (UNEP/CBD/BS/COP-MOP/3/INF/1^1^). In Decision BSIII/11^1^, after the report from the Rome AHTEG, the Parties called on the Secretariat to collect existing information on risk assessment and make it available. The importance of this request, however, was lost when it became subsumed in the work that ensued on the development of guidance on risk assessment in the coming years.

A decision was later made at COP-MOP4 to form another AHTEG and an online forum and to begin work to develop guidance. The original terms of reference for the AHTEG was laid out in the annex to the COP-MOP4 decision on risk assessment (Decision BSIV/11^1^):

‘*Develop a “roadmap”, such as a flowchart, on the necessary steps to conduct a risk assessment in accordance with Annex III to the Protocol and, for each of these steps, provide examples of relevant guidance documents;’*

‘*Prioritize the need for further guidance on specific aspects of risk assessment and define which such aspects should be addressed first, taking also into account the need for and relevance of such guidance, and availability of scientific information;’*

‘*Define an action plan to produce modalities for development of the guidance documents on the specific aspects that were identified as priorities.’*

The “prioritization of topics” for further guidance, i.e., “specific aspects” of risk assessment in addition to the Roadmap, was part of the AHTEG's original mandate. The earlier, 2005 Rome AHTEG had also acknowledged that there may be a need for specific guidance in the future, and in discussing specific gaps in existing approaches and guidance materials, the Rome AHTEG listed a wide range of examples of specific areas where “existing guidance may not be sufficient” (UNEP/CBD/BS/COP-MOP/3/INF/1^1^). The list described what were then new techniques, product concepts, and new or less familiar issues to regulators and risk assessors. Over time, a similar reasoning was applied in proposing more new topics for guidance. The AHTEG, at its first meeting after COP-MOP4, engaged in a “priority setting exercise” which resulted in a list of 14 “prioirtized topics for the development of guidance” (Annex II of UNEP/CBD/BS/COP-MOP/5/INF/13^1^). The methods to rank the topics was not described in the report from the AHTEG meeting, and it seemed to be based primarily on the number of requests for guidance on a certain topic by some Parties. The AHTEG then defined “*an action plan to produce modalities for development of the guidance documents”* on the topics, which apparently was to work in subgroups and actually draft guidance on the top priority topics, in parallel with the Roadmap, with input from the online forum.

First drafts of the Roadmap as well as the additional guidance on stacked genes, abiotic stress and mosquitos were developed in the interim between COP-MOP4 and COP-MOP5 (UNEP/CBD/BS/COP-MOP/5/INF/13^1^; UNEP/CBD/BS/COP-MOP/5/INF/15^1^), and additional guidance on trees and monitoring was added in the interim between COP-MOP5 and COP-MOP6 (UNEP/CBD/BS/COP-MOP/6/INF/10^1^) (see Figure 1). It was also between COP-MOP5 and COP-MOP6 that the AHTEG decided to organize the guidance into three parts, placing the most recently developed additional guidance on Monitoring into a “Part” separate from the other additional guidance. Before COP-MOP8, the AHTEG prioritized two additional topics for guidance: “living modified fish” and “synthetic biology,” and developed outlines for guidance on these topics. These outlines were presented in the report to COP-MOP8 (UNEP/CBD/BS/COP-MOP/8/8/Add.1^1^) with the last version of the Guidance, where there was no decision to pursue these two additional topics.

Regarding the Roadmap section of the Guidance specifically, in the analysis from the open-ended online forum discussions presented to COP-MOP5 (BS/COP-MOP/5/INF/12^1^), after the first 2 years of work on the Guidance, the intent of the Roadmap specifically was further elaborated as follows:

‘*… the roadmap should be a practical guide to assist risk assessors and decision makers on how to implement the provisions set out in the Annex III of the Protocol.’*

‘*The roadmap is envisaged to provide additional detailed guidance on how to conduct risk assessment of LMOs …’*

‘*Furthermore, the roadmap is to serve as a reference to guidance materials that are relevant to each step or point to consider.’*

After the discussions that took place at COP-MOP5, in the COP-MOP5 decision (Decision BSV/12^1^) the purpose of the Guidance appeared to shift noticeably from a “practical” and “detailed” guidance on risk assessment to a reference document:

“*… its objective is to provide a reference that may assist Parties and other Governments in implementing the provisions of the Protocol with regards to risk assessment, in particular its Annex III …”*

In the latest version of the Guidance (BS/COP-MOP/8/8/Add.1^1^), there is a further attempt to emphasize the more general applicability of the Roadmap by the addition of text in the “background” section of the Roadmap itself:

‘*The Roadmap introduces basic concepts of risk assessment rather than providing detailed guidance for individual case-specific risk assessments. In particular, the “elements for consideration” listed in the Roadmap may need to be complemented by further information during an actual risk assessment*.’

The Guidance in its current form may “provide a reference for risk assessment” of LMOs as stated in the COP-MOP5 decision, and “introduce basic concepts” as stated in the background to the Roadmap in the most recent version; yet it may not be useful as a harmonized “roadmap” or “guide” to assist risk assessors, as seemed to be its original intent. Rather than guidance based on an agreement about what is actually done in risk assessments based on experience, the Guidance attempted to represent many, varying opinions expressed by the participants of the AHTEG and the online forum about what “should be done” in risk assessment. In the note by the Executive Secretary on Risk Assessment and Risk Management for COP-MOP8 (UNEP/CBD/BS/COP-MOP/8/8^1^), it states: “*The AHTEG endeavored to reconcile the different views coming from the Online Forum by following an inclusive approach to explore all possibilities to reach a middle ground on the outstanding issues*.” In fact, the resulting Guidance is a compromise document that attempts to merge some irreconcilable points of view, including views on many non-technical issues, without maintaining a connection to the source of those different views. The reports from the AHTEG meetings frequently indicate where the AHTEG had “agreed,” when in fact there was compromise necessitated by the need to keep the process moving, and secured without consensus among experts.

## Misplaced Expertise to Develop the Guidance

### Party Members of the AHTEG

In order to understand the challenge for reaching a meaningful agreement in developing the Guidance, it is useful to consider the history of the AHTEG, its composition and membership. There were two phases of the AHTEG (actually two separate AHTEGs with some overlap of individuals as members) that worked on the Guidance, from 2008 to 2012 and 2012 to 2016 (see Figure 1 and Table 1; a list of all AHTEG members can be found on the Biosafety Clearing House^1^). The “Party members” of the AHTEG were individual experts nominated by their Parties and selected by the Executive Secretary, more-or-less in accordance with the consolidated modus operandi of the Subsidiary Body on Scientific, Technical, and Technological Advice (SBSTTA) (see Box 3 and Box 4). Therefore, the composition of the AHTEG as selected from the list of nominated experts attempted to consist of equal representation from each of the five regional groups of the United Nations “*with due regard to geographical representation, gender balance, and to the special conditions of developing countries …*” In the end, a total of 26 countries were represented in one or both phases of the AHTEG; 16 of these were considered developing countries or economies in transition^2^ as of December 2016 when the Guidance was presented to COP-MOP8. The EU as a “Party” was not represented on the AHTEG, although six of the EU member state countries were Party members on the AHTEG over the two phases (Table 1).

**Table 1:** Composition of the AHTEG on risk assessment and risk management.

**Region/party**		**AHTEG 1**		**AHTEG 2**		
		**MOP4-MOP5 2008-2010**	**MOP5-MOP6 2010-2012**	**MOP6-MOP7 2012-2014**	**MOP7-MOP8 2014-2016**	**No. of crops approved for cultivation^**+**^**
**AFRICA**						
Egypt/Mauritania^*^	D	x	x	x	x	1
Nigeria (1)	D	x	x			0
Nigeria (2)	D	x	x			-
Niger	D	x	x			0
South Africa	D			x	x	3
Zimbabwe	D			x	x	0
Kenya	D				x	0
**ASIA &PACIFIC**						
China^#^	D	x	x	x	x	8
Malaysia (1)	D	x	x	x	x	1
Malaysia (2)	D	x	x			-
Japan		x	x	x	x	10
India	D				x	1
**EASTERN EUROPE**						
Croatia (EU)		x	x	x	x	^#^
Republic of Maldova	D	x	x	x	x	0
Slovenia (EU)		x	x		x	^#^
Belarus	D			x	x	0
**LATIN AMERICA & CARIBBEAN**						
Brazil	D	x	x			4
Cuba	D	x	x			1
Mexico^#^	D	x	x	x	x	3
Colombia	D			x	x	5
Honduras	D			x	x	1
Antigua & Barbuda					x	0
**WESTERN EUROPE & OTHERS**						
Austria (EU)		x	x	x	x	^#^
Germany (EU)		x	x	x		^#^
Netherlands (EU)		x	x			^#^
Norway		x	x		x	1
Finland (EU)				x	x	^#^
New Zealand					x	0
**OTHER (NON-PARTY) GOVERNMENTS (OBSERVERS)**						
Australia		x	x	x	x	4
Canada		x	x	x	x	8
United States		x	x			17
Argentina				x	x	5
**OTHER ORGANIZATIONS (OBSERVERS)**						
Bayer Crop Science		x	x	x	x	
Monsanto Company		x	x	x	x	
University of Canterbury		x	x	x	x	
Institute de Estudios Ecologists		x	x			
Federation of German Scientists		x	x			
Public Research & Regulation Initiative		x	x			
College of the Atlantic				x	x	
Flinders University				x	x	
University of Minnesota				x	x	

*“D” denotes developing country or economy in transition status according to United Nations (2018)^2^*.

* *The same expert individual was nominated by Egypt for the first AHTEG and by Mauritania for the second AHTEG*.

+ *No. of different crops approved according to the ISAAA GM Crop Approval Database (see Table 2) as of COP-MOP8 in 2016*.

# *The ISAAA database lists 3 crops approved by the EU (see Table 2), not by the individual EU member states*.

Box 3 **Consolidated modus operandi of the subsidiary body on scientific, technical and technological advice of the convention on biological diversity, paragraph 18 a,b, and e.**

**Description of** ***ad hoc* technical expert groups**

18. A limited number of ad hoc technical expert groups on specific priority issues on the programme of work of the Conference of the Parties may be established under the guidance of the Conference of the Parties, as required, for a limited duration, to provide scientific and technical advice and assessments. The establishment of such ad hoc technical expert groups would be guided by the following elements:
  (a) The ad hoc technical expert groups should draw on the existing knowledge and competence available within, and liaise with as appropriate, international, regional and national organizations, including non-governmental organizations and the scientific community, as well as indigenous and local community organizations and the private sector, in fields relevant to this Convention;
  (b) The Executive Secretary, in consultation with the Bureau of the Subsidiary Body on Scientific, Technical and Technological Advice, will select scientific and technical experts from the nominations submitted by Parties for each ad hoc technical expert group. The ad hoc technical expert groups shall be composed of no more than fifteen experts nominated by Parties competent in the relevant field of expertise, with due regard to geographical representation, gender balance and to the special conditions of developing countries, in particular the least-developed and small island developing States, and countries with economies in transition, as well as a limited number of experts from relevant organizations, depending on the subject matter. The number of experts from organizations shall not exceed the number of experts nominated by Parties;
  (e) Reports produced by the ad hoc technical expert groups should, as a general rule, be submitted for peer review;

Box 4 **Annex of decision BS-IV/11 on risk assessment, paragraph 1a-b.**

**Modality of work and the terms of reference for the AHTEG on RA&RM**

1. The ad hoc Technical Expert Group (AHTEG) on Risk Assessment and Risk Management shall:
  (a) Include experts selected on the basis of their expertise on the issues relevant for the mandate of the Group, based on a standardized common format for submission of CVs from experts nominated by Parties, respecting geographical representation, in accordance with the consolidated modus operandi of the SBSTTA of the Convention on Biological Diversity (decision VIII/10 of the Conference of the Parties, annex III);
  *(b) Include observers in accordance with the rules of procedure for meetings of the Conference of the Parties serving as the meeting of the Parties to the Protocol*.

**Decision BS-VI/12 on Risk Assessment, Paragraph 8a-c**

**Request to the Executive Secretary**

8. Requests the Executive Secretary to:
  (a) With a view to achieving a balance of current and new members, select experts for the new AHTEG, in consultation with the Bureau of the Conference of the Parties serving as the meeting of the Parties to the Protocol, in accordance with paragraph 18 of the consolidated modus operandi of the Subsidiary Body on Scientific, Technical and Technological Advice of the Convention on Biological Diversity (decision VIII/10, annex III);
  (b) Invite other Governments and relevant international organizations to participate in the open-ended online forum;
  (c) Ensure that the participation of experts nominated by other Governments and relevant organizations to the open ended online forum and AHTEG is in accordance with rules 6 and 7 of the rules of procedure for meetings of the Conference of the Parties serving as the meeting of the Parties to the Protocol;

The relevance of the Party nominee's expertise “*on the issues relevant for the mandate of the group*” was assessed by the CBD Executive Secretary in order to select these AHTEG members (see Box 4). Although the AHTEG members were clearly valued as experts in their fields by the national focal points by who they were nominated, this did not necessarily equate with experience conducting actual cases of risk assessment with LMOs in their countries. In fact, only a small subset of the Party countries have conducted risk assessments for commercial production (see Table 2 for a list), which could be considered an indication of a Party's “experience” with ERA most like the risk assessment called for in the Cartagena Protocol. (“Commercial Production” is the terminology used here, as it is in the third national reports^3^ on implementation of the Protocol, to distinguish the scope of the risk assessment from Field Trials; Contained Use; Food; Feed; Processing. This may also be referred to as “for cultivation” as in the ISAAA database, among other terminology such as “deregulation” or “general release” used in some countries^4^.) In fact, many countries that are Party to the Protocol do not yet have biosafety frameworks in place to regulate biotechnology.

**Table 2:** Countries with crops approved for cultivation, and the crops approved in each (Taken from the ISAAA GM Approval Database^a^).

**Crops approved for cultivation**				
			**As of COP-MOP8 in December 2016**	**After COP-MOP8 in December 2016**
**PARTY**				
	D	Bangladesh	Eggplant	
A	D	Bolivia	Soybean	
A	D	Brazil	Bean, cotton, maize, soybean	Eucalyptus, sugarcane
	D	Burkina Faso	Cotton	
A	D	China	Cotton, maize, papaya, petunia, poplar, rice, sweet pepper, tomato	
A	D	Colombia	Carnation, cotton, maize, rose, soybean	
	D	Costa Rica	Cotton, soybean	
	D	Cuba	Maize	
A	D	Egypt	Maize	
	D	Ethiopia		Cotton
A		European Union	Carnation, maize, potato	
A	D	Honduras	Maize	
A	D	India	Cotton	
	D	Indonesia	Sugarcane	
	D	Iran	Rice	
A		Japan	Alfalfa, canola, carnation, cotton, maize, papaya, rice, rose, soybean, sugarbeet	
A	D	Malaysia	Carnation	
A	D	Mexico	Alfalfa, cotton, soybean	
	D	Myanmar	Cotton	
A	D	Nigeria		Cotton
		Norway	Carnation	
	D	Pakistan	Cotton	Maize
	D	Panama	Maize	
	D	Paraguay	Cotton, maize, soybean	
	D	Philippines	Maize	
A	D	South Africa	Cotton, maize, soybean	
	D	Sudan	Cotton	
	D	Swaziland		Cotton
	D	Uruguay	Maize, soybean	
	D	Vietnam	Maize	
**OTHER (NON-PARTY) GOVERNMENTS**				
O	D	Argentina	Cotton, maize, soybean	alfalfa, potato
O		Australia	Canola, carnations, cotton, rose,	safflower
O		Canada	Alfalfa, apple, canola, flax, maize, potato, soybean, sugarbeet	
	D	Chile	Canola, maize, soybean	
O		United States	Alfalfa, apple, canola, chicory, cotton, flax, maize, papaya, plum, potato, rice, rose, soybean, squash, sugarbeet, tobacco, tomato	creeping bentgrass,

a *http://www.isaaa.org/gmapprovaldatabase/default.asp as of Sept. 29, 2018*.

*“D” denotes developing country status according to United Nations (2018)^2^*.

*“A” denotes a Party member and “O” denotes observer member of the AHTEG on Risk Assessment and Risk Management*.

If experience with approvals for commercial production is an indication of a Parties' experience with actual cases of risk assessment, then much of this expertise may have been missing among the Party members of the AHTEG. By the time the Guidance was presented at COP-MOP8, 31 countries in the world (26 Parties; five non-Parties) and the EU had approved crops for commercial production, i.e., “for cultivation” according to the ISAAA database on GM (genetically modified) crop approvals. Since then, three more countries (Ethiopia, Nigeria, Swaziland) have also approved a crop for commercial production. Table 2 shows these 34 countries and the EU, and the crops that have been approved in each. Most of these countries fall into the category of a “developing country”^2^. Of the 26 Party countries that had approved GM crops for commercial production, twelve were represented on the AHTEG; fourteen countries that had approved GM crops for commercial production were not represented on the AHTEG, as shown in Table 2.

The number of different crops approved by each country represented on the AHTEG is also shown in Table 1. Of the Parties represented on the AHTEG, 12 had approved one or more crop, and eight had approved none. This does not include the six EU member states represented on the AHTEG; although the EU is listed in the ISAAA database for three crop approvals (Table 2), the database does not bring up any approvals by individual member states. This is because approvals for commercial production are made at the level of the EU following a review by the European Food Safety Authority (EFSA), and not by individual member states; Therefore, the level of experience with actual cases of risk assessment varies among experts from individual EU member states, including the six EU experts that were Party members of the AHTEG.

### Observer Members of the AHTEG

In addition to the “Party” expertise on the AHTEG, experts nominated by non-Party governments were also selected to participate in the AHTEG as “observers,” as were experts from other relevant organizations including industry, academia, and other non-government organizations (NGOs) (see Table 1). The description of an AHTEG in paragraph 18(a) from the consolidated modus operandi of the SBSTTA (Box 3) does indicate that an AHTEG should draw on knowledge and competence from Party experts, as well as experts in the field from ‘*international, regional and national organizations, including non-governmental organizations and the scientific community, as well as indigenous and local community organizations and the private sector*.’ Paragraph 18 of the modus operandi of the SSBTTA does not specify the level of participation from observers, nor does it refer to the rules of procedure for meetings of the Conference of the Parties to the CBD.

However, in the case of the AHTEG on Risk Assessment and Risk Management, and in the open ended online forum, language was included to clearly specify, in the terms of reference in the annex of Decision BSIV/11^1^ and as a request to the Executive Secretary in the main text of Decision BSVI/12^1^ (see Box 4), that participation of observers would be in accordance with the “rules of procedure” for meetings of the Conference of the Parties to the Convention on Biological Diversity and its protocols. (The “rules of procedure” can be found on the Convention on Biological Diversity website: www.cbd.int/convention/rules.shtml). According to the “rules of procedure”: *observers* [represented at meetings of the Conference of the Party] *may participate without the right to vote in the proceedings of any meeting in matters of direct concern to the body or agency they represent …'* In extending this to members of an AHTEG, this meant that experts from non-Parties and other observers were allowed to attend the face-to-face meetings of the AHTEG and participate in those discussions, as were non-Party and others allowed to contribute posts to the online forum, but these observers did not participate in discussions or decisions on recommendations of the expert group to the COP-MOP. In the case of the AHTEG, observers were at times not even allowed to listen to the discussion on the recommendations among the Party members of the AHTEG.

The reference to the “rules of procedure” in the decisions by the COP-MOP referred to above, which clearly limits participation of non-Party and other experts in the case of this particular AHTEG, may have been considered important by some in order to prevent a conflict of interest, for any purpose, by perceived non-Party proponents or antagonists of biotechnology. At the same time, it almost certainly also limited the AHTEG's ability to develop practical and useful guidance taking into account past and present experiences with LMOs. Most of the global experience with risk assessment of LMOs can be found among the “observers,” including the non-Party governments that have issued the vast majority of the approvals for biotech products (Table 2). Although the US signed but did not ratify the Convention on Biological Diversity and therefore cannot be a party to the Protocol, as the leading adopter of GM crop applications of biotechnology, the US has participated to the full extent possible as an “observer” in the discussions under the Protocol since the earliest negotiations, as have Canada, Australia, and Argentina, which are also not Party to the Protocol.

### The Open-Ended Online Forum

The open-ended online forum on risk assessment and risk management was meant to ensure that multiple experts from Party and non-Party countries, and other organizations could contribute to the discussion and be used by the AHTEG in their deliberations, but this also had limitations, including the restrictions of the rules of procedure (see the text from Decision BS-VI/12^1^ paragraph 8c in Box 4). The list of registered online forum participants and all of the online forum discussions can be found in their entirety on the Biosafety Clearing House^1^. There were ~300 individuals enrolled in the online forum with a wide range of expertise, although a much smaller number of these individuals regularly participated in any given forum discussion; ~75% of those enrolled were individuals nominated by Parties, ~10% nominated by non-Party governments, and the rest from “other organizations.”

An example of the online forum participation comes from the last online forum discussion that took place before COP-MOP8 (April 25-May 9 2016). The topic of this discussion was “Feedback on the Proposed Revisions to the Guidance.” This was an important online forum discussion because it was the only opportunity to provide feedback by individuals not on the AHTEG, to the AHTEG's proposed revisions based on the comments from the testing of the Guidance (discussed in more detail later). Instead, the feedback requested in this forum discussion was strictly limited to certain revisions and comments on the whole document were not invited. The discussion was open for 2 weeks (which was typical), with six posts coming in the first week and 48 coming in the second week. These 54 posts came from 29 individuals: 14 nominated from eight Parties (two who were members of the AHTEG), three nominated from three non-Parties, and 12 nominated from ten other organizations, including several who were members of the AHTEG. While a number of posts in this forum discussion were supportive of the Guidance, a number also shared frustration with the limited ability the forum presented for input on the Guidance.

The online forum was commended by the COP-MOP in its COP-MOP5 decision (Decision BSV/12^1^) as an innovative method and efficient means to maximize the use of limited resources. It did provide an opportunity for participation by a large group of experts with broad and diverse backgrounds and experiences, with varying motivations to participate, including the non-Party governments, the biotech industry, academics, and non-government organizations, some with clear pro- or anti-biotech agendas. However, the requests for input in the online forum over the years were generally narrowly limited to specific points determined by the CBD secretariat, and although this may have been necessary for the functioning of the forum, it was not clear how the input from the online forum on these specific points was ultimately used, by the AHTEG or in other ways, to shape the Guidance. Although the online forum was a good idea in theory and did provide an opportunity for more experts to voice an opinion, in practice it did not offer an effective tool to develop or improve the Guidance.

### Weighing Expert Input vs. Party Input

It was not always clear whether the members of the AHTEG or Online Forum participants, from Parties or others, were meant to be contributing to the discussions based on their own experiences as experts with risk assessment, or on behalf of the political positions of the governments or organizations that nominated them. In the latter case, particularly in following the “rules of procedure” of the Convention, the discussions were bound to and did become more like the negotiations of the Parties and less like an expert consultation. It would seem from the description of an AHTEG in the consolidated modus operandi of the SBSTTA (paragraph 18(a) in Box 3), that an AHTEG should be seeking “expert” input, rather than “Party” input. Yet, the discussions of this AHTEG and the online forum often appeared to be “Party”-driven, rather than “expert”-driven, with a tally of Party vs. other expert opinions on each side of an issue.

In the final deliberation, after an exhausting eight years, the “Party members” of the AHTEG, without the “observer members” of the AHTEG which was according to the “rules of procedure” as specified in the COP-MOP decisions (Box 4), “unanimously” agreed to recommend endorsement of the Guidance to the COP-MOP (UNEP/CBD/BS/COP-MOP/8/INF/2^1^). It was made clear at COP-MOP8, however, that a decision by the “Party members” of the AHTEG was not the same as the decision by the Parties at the COP-MOP. Although the priority to Parties on the AHTEG meets with the rules of procedure of meetings of the Convention and its protocols, it does not clearly align with the role of an AHTEG as set forth in the consolidated modus operandi of the SBSTTA, and it was apparently not an effective means to develop technical guidance based on expert input. It is always a challenge to separate political discussions from technical issues in risk assessment and regulation of biotechnology (Hokanson et al., 2018). The experience with this AHTEG further demonstrates what should be obvious, that it is not practical, if even possible, to “negotiate” the contents of a technical guidance document.

## Misguided Testing of the Guidance

### The Testing Process

More difficulties for the development of the Guidance were encountered in the testing that was conducted between COP-MOP6 and COP-MOP8. When the first draft of the Guidance was presented at COP-MOP5 the Parties called for ‘*further scientific reviewing and testing to establish its overall utility and applicability*’ (Decision BSV/12^1^). In response, after COP-MOP5 there was a further round of revisions by the AHTEG and the online forum. At COP-MOP6, the Parties commended the progress on the Guidance, and called for the Guidance to be ‘*tested nationally and regionally for further improvement in actual cases of risk assessment and in the context of the* [Protocol]' (Decision BSVI/12^1^). In response after COP-MOP6, the first AHTEG was brought to a close, and a reconstituted AHTEG was established (see Figure 1 and Table 1), and Parties, other Governments, and other organizations were encouraged ‘*through their risk assessors and other experts who are actively involved in risk assessment, to test the Guidance in actual cases of risk assessment*’ and submit the results to the Biosafety Clearing House.

A notification for the testing of the Guidance (SCBD/BS/CG/MPM/DA/82041^1^) described the process for the testing in broad terms, including the use of a specified form (‘*The Questionnaire for Reporting Results of the Testing of the Guidance on Risk Assessment of Living Modified Organisms*’^1^). The methodology to employ for conducting the test “in actual cases of risk assessment” was not specified beyond a recommendation to identify an “actual case” to consider. There was no recommendation on how “risk assessors” should be identified, and a description of the credentials of the testers or description of the testing methodology employed by the testers was not requested with the submissions. Thus, it was such that tests were apparently conducted in any number of undefined, different ways. The form simply asked the testers to rate the six parts of the Guidance (Box 2) on a scale from 1 (Strongly Agree) to 5 (Strongly Disagree) for each of four criteria: (1) “practical,” (2) “useful,” (3) “consistent with the protocol,” and (4) “takes into account past and present experiences with LMOs.” For each of the sections rated there was also a space to suggest specific improvements, and a space at the end of the questionnaire to “*provide additional feedback regarding the testing of the Guidance*.”

### The Results of the Testing

The ‘*individual submissions*’ (filled questionnaires) from all of the participants in the testing can be found on the Biosafety Clearing House^1^, and a report on the results of the testing was shared at COP-MOP7 (UNEP/CBD/BS/COP-MOP/7/INF/3^1^). Forty-three of the 171 Parties to the Protocol (25% of all Parties), three non-Party governments, and ten ‘other organizations’ participated in the testing (see Tables 3A,B). All of these participants tested the Roadmap section (Part I) of the Guidance (see Box 2); many participants submitted test results on the Roadmap only, while the other sections were tested only by some and not by others. Of the 43 Parties that participated, 28 are considered “developing countries,”^2^ as described in the report on the results.

The participation of these developing country Parties held significance in the Secretariat's analysis of the results, presumably because the Protocol (in Article 22) calls for capacity building in biosafety for the purpose of effective implementation of the Protocol in developing country Parties and in Parties with economies in transition. The Strategic Plan for the Cartagena Protocol for the period 2011–2020 (Decision BS-V/16, Annex I), coincidentally agreed to by the COP-MOP after the work of the AHTEG had begun, includes risk assessment and risk management as part of its capacity building objectives, and indicators to measure progress include measures of Parties that are using the developed technical guidance and that are of the opinion that the technical guidance is sufficient and effective. Thus, it seemed the Secretariat viewed “developing countries” that are the target of capacity building as an important group for which to measure the level of agreement with the criteria in the testing.

In the report, the results are shown in a bar graph as the ‘*overall level of agreement that* [the Guidance] *is practical, useful, consistent with the Protocol, and takes into account the past and present experiences with LMOs*’ averaged across the ratings for the four distinct criteria within certain groupings (i.e., All Parties, Developing Country Parties, Other Governments, and Organizations). A series of graphs also showed these groupings for each of the criteria independently and for the different sections of the Guidance. All of those graphs show that average scores from the developing country parties were equal to or slightly higher than from all Parties, and both of these groups' scores were considerably higher than the average score from the three non-Party governments that participated in the testing. Although an interpretation of these results as reported to COP-MOP7 may arguably not be particularly meaningful, if interpreted as a measure of the level of “agreement” that the Guidance meets the criteria, the relative scores among these groups could be an indication that the developing country Parties “agree” the most that the Guidance is “practical,” “useful,” “consistent with the Protocol,” and “takes into account past and present experience.” Likewise, it could be surmised that non-Party governments “agree” the least.

**Figure 2:**
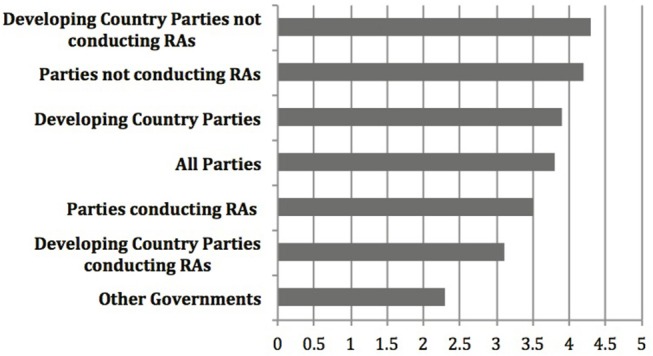
Overall level of agreement that the Roadmap is practical, useful, consistent with the Cartagena Protocol on Biosafety, and takes into account past and present experience with LMOs, based on the results of the testing of the Guidance as gathered by the CBD Secretariat, where 1 is strongly disagree and 5 is strongly agree (The results of the testing can be found at https://bch.cbd.int/protocol/testing_guidance_RA.shtml).

Yet, relative scores among other groupings not considered as part of the report to COP-MOP7, could indicate something different. Most important is the notable difference between ratings provided by countries (Party, Non-Party, or Developing) that have experience with conducting risk assessments and those that don't. Figure 2 shows the number scores for the testing, specifically on the Roadmap section of the Guidance (here the focus is on the Roadmap because it is the core of the Guidance and the section tested by all participants) for additional groupings averaged across all four criteria. This includes three of the groupings included in the report (All Parties, Developing Country Parties, Non-Party Governments), and four additional groupings. These are “Parties that have conducted risk assessments” for commercial production and “Parties that have not conducted risk assessments” for commercial production (many of these do not yet have biosafety frameworks) according to the third national reports^3, 4^. Developing countries can be further grouped into “Developing Country Parties that have” or “Developing Countries that have not” conducted risk assessments for commercial production. Figure 2 demonstrates that there is more disparity between the higher average score from Parties who *have not* conducted risk assessments for commercial production (4.1) and the lower score from Parties who *have* conducted these risk assessments (3.6), and even more so between developing country parties who *have not* conducted these risk assessments (4.3), and those who *have* (3.1).

These trends from the results based on the number scores of the testing seem to indicate that Parties, including developing country Parties, who have more experience with risk assessment, rated the Roadmap lower across all criteria than did those with less experience. If this is the case, it stands to reason that the non-Party governments, who presumably have the most experience conducting risk assessments on LMOs, agreed the least that the testing met the criteria of “useful,” “practical,” “consistent with the Protocol,” and “takes into account past and present experience with LMOs.” The relatively lower scores among Parties who have experience compared to those with less experience may tell us more about the utility and applicability of the Roadmap than does the relative score of developing countries compared to all Parties that was central in the analysis from the Executive Secretary in the report to COP-MOP7. Regardless, these number scores can only tell us how the testers rated the Guidance against the testing criteria, and tell us very little about the utility and applicability of the Guidance in ‘*actual cases of risk assessment*,’ which was the stated objective of the testing according to Decision BSVI/12^1^.

### The Revisions Based on the Testing

However the trends are interpreted, it would be imprudent to only consider the number scores as an indication of the practicality or usefulness of the Guidance. At COP-MOP7, where the report from the testing (UNEP/CBD/BS/COP-MOP/7/INF/3^1^) was presented to the Parties, it was decided that the Guidance should still be revised and improved ‘*on the basis of the feedback provided through the testing with a view to having an improved version of the Guidance by MOP8*’ (Decision BS-VII/12^1^). More than 775 comments were submitted by participants in the testing on the questionnaires along with the ratings; 488 of these comments were on the Roadmap (Part I) of the Guidance alone. The numbers of comments submitted by the participants on the Roadmap section only are shown in Tables 3A,B, and it should be noted that in general participants who gave the Roadmap (as with the Guidance) lower scores than those who gave higher scores also submitted more comments.

**Table 3A:** Results from participants in the testing of the Roadmap (Part I) of the ‘Guidance on Risk Assessment of LMOs developed by the AHTEG under the Cartagena Protocol on Biosafety, and the answers by the Party countries to questions on risk assessment (see Box 6) in the third national reports3 on implementation of the Protocol, by countries that are currently conducting risk assessments for commercial production.

**Q89. YES—Conducting any risk assessments**					
**Q90. YES—Conducting Risk Assessments for Commercial Production**					
		**Q85a. Using any guidance**	**Q86. Using AHTEG guidance**	**Roadmap testing results**	
				**Avg. score all criteria**	**Number of comments**
**Party**					
Norway		Yes	Yes	5	5
Cuba	D	Yes	Yes	4.75	2
Austria (EU)		Yes	No	4.75	15
Egypt	D			4.75	4
Czech Republic (EU)		Yes	Yes	4.5	7
Slovenia (EU)		Yes	No	4.25	0
Spain (EU)		Yes	Yes	4.25	1
Costa Rica	D	Yes	Yes	4	4
European Union^*^		Yes	No	4	7
Germany (EU)		Yes	No	4	21
Portugal (EU)		Yes	No	4	0
Mexico	D	Yes	No	4	19
South Africa	D	Yes	No	4	12
Italy (EU)		Yes	No	3.75	1
Uruguay	D	Yes	No	3.75	2
VietNam	D	Yes	No	3.5	1
Belgium (EU)		Yes	No	3	5
Colombia	D	Yes	No	2.5	11
Netherlands (EU)		Yes	No	2.25	7
India	D	Yes	No	2.25	48
Brazil	D	Yes	No	2	27
Honduras	D	Yes	No	2	6
Japan		Yes	No	2	25
Philippines	D	Yes	No	2	1
Average				3.55	9.6
**Other (Non-Party) Governments**					
Canada				2.5	21
United States				2.25	10
Australia				2	50
Average				2.13	27

*“D” denotes developing country or economy in transition status according to the United Nations (2018)^2^*.

* *The European Union participates in the COP-MOPs as a Party, as do the individual member states*.

**Table 3B:** Results from participants in the testing of the Roadmap (Part I) of the “Guidance on Risk Assessment of LMOs” developed by the AHTEG under the Cartagena Protocol on Biosafety, and the answers by the Party countries to questions on risk assessment (see Box 6) in the third national reports^3^ on implementation of the Protocol, by countries that are currently not conducting risk assessments for commercial production or not conducting any risk assessments.

**Q89. YES—Conducting any risk assessments**					
**Q90. NO—Conducting Risk Assessments for Commercial Production**					
		**Q85a. Using any guidance**	**Q86. Using AHTEG guidance**	**Roadmap testing results**	
				**Avg. score all criteria**	**Number of comments**
**Party**					
Bolivia	D	No	No	4.5	13
Belarus	D	Yes	Yes	4.5	18
Denmark (EU)		Yes	No	4	3
Turkey	D	No	No	3.5	2
New Zealand		Yes	Yes	2.25	2
Malaysia	D	Yes	Yes	2.25	25
El Salvador	D	Yes	Yes	2	19
**Q89. No—Not Conducting Any Risk Assessments**					
Libya^*^	D	–	–	5	0
Mauritania	D	No	No	5	0
Niger	D	No	No	5	0
Republic of Moldova	D	No	Yes	5	5
Syria^*^	D	–	–	5	0
Tajikistan	D	No	No	5	1
Yemen	D	No	Yes	5	0
Liberia	D	No	No	4.75	1
Bosnia	D	Yes	No	4.25	0
Hungary (EU)		Yes	No	4.25	5
Georgia	D	Yes	No	4	0
Peru	D	No	No	3.5	15
Average				4.14	5.7
**Other Organizations**					
Eco-Tiras				5	6
GenOk-Center for Biosafety				5	4
Int’l Association for Human&Animal Health Improvement				5	0
State University Maldova				5	4
Third World Network				5	8
Academy of Science Maldova				4.75	5
ENCA EPA				4.75	9
Friends of the Earth-Ukraine				4.5	3
Public Research & Regulation Initiative				2	16
Global Industry Coalition				1.75	5
Average				4.275	6.7

*“D” denotes developing country or economy in transition status according to the United Nations (2018)^2^*.

* *Did not submit third national reports by the time of COP-MOP8*.

In the decision from COP-MOP7, the Parties established a mechanism for the AHTEG to revise and improve the Guidance on the basis of the feedback, as described in some detail in paragraph 1 of the terms of reference for the online forum and AHTEG in the Annex to Decision BS-VII/12^1^ (see Box 5). However, the “streamlining” of the comments outlined in paragraph 1(c) of the methodology was not done by the AHTEG as described, but by a subgroup of five AHTEG members (experts nominated from China, Finland, Mexico, Republic of Moldova, and Zimbabwe, selected from the AHTEG at its last face-to-face meeting *before* COP-MOP7 (BS/COP-MOP/7/10/Add.2^1^). This subgroup decided which comments would be “taken on board.” A record of the “Subgroup Discussions (2014–2016)” can be found on the Biosafety Clearing House^1^, and a document with the justifications for the actions taken by the subgroup on every comment submitted in the testing of the Guidance was provided to COP-MOP8 (BS/COP-MOP/8/INF4^1^).

Box 5 **Annex of decision BS-VII/12, paragraph 1a-d.**

***AHTEG mechanism to improve and revise the Guidance***

1.Taking into account the results of the testing process, established in decision BS-VI/12, the Guidance on Risk Assessment of LMOs shall be revised and improve in accordance with the following mechanism:
  (a)After the seventh meeting of the COP-MOP, the Secretariat will group the original comments provided through the testing of the Guidance. The grouping will be done in the form of a matrix based on the following categories: statements that do not trigger changes; editorial and translational changes; suggestions for changes without a specified location in the Guidance; and suggestions for changes to specific sections of the Guidance (sorted by line numbers);
  (b)The AHTEG shall review the grouping of comments done by the Secretariat and work on the suggestions for changes;
  (c)The AHTEG shall streamline the comments by identifying which suggestions may be taken on board and providing justification for those suggestions that may not be taken on board. The AHTEG will also provide concrete text proposals for the suggestions to be taken on board with a justification where the original suggestion was modified;
  *(d)The Open-ended Online Forum and the AHTEG shall subsequently review all comments and suggestions with a view to having an improved version of the Guidance for consideration by the COP-MOP at its eighth meeting*.

Although the work of the subgroup was completely transparent by making their assessments and justifications available for viewing, it would be incorrect to assume that these justifications were the work of the entire AHTEG and the Online Forum. Neither the AHTEG nor the Online Forum had an opportunity to discuss many of the decisions by the subgroup about whether or not to “take comments on board.” This process did result in numerous and significant changes to the Guidance by the time it was presented at COP-MOP8 for the Parties to consider, although many of the concerns raised in the feedback to the testing were still not addressed in the most recent revised version. At COP-MOP8, unfortunately, a number of Parties did not feel there had been an opportunity to consider whether these changes resulted in an “improved” guidance, resolving some of the more serious concerns with the Guidance that had been expressed over the years in the on-line forum and AHTEG discussions, or as a result of and in the comments from the testing. The outcome after this lengthy and arduous process employed to test the Guidance and revise it based on the results of the testing seems to indicate that unfortunately the testing process missed its mark.

## A Closer Look at Experience in Relation to the Guidance

### Experience Based on the Third National Reports

The third national reports^3^ submitted by the Parties on the implementation of the Protocol shed even more light on the relationship between experience with risk assessment and the development and testing of the Guidance. The reports included answers to questions regarding risk assessment of LMOs in relation to the use of guidance, including “the Guidance.” (The relevant questions related to risk assessment and the Guidance are shown in Box 6). In the official meeting document on Risk Assessment and Risk Management (UNEP/CBD/BS/COP-MOP/8/8^1^) prepared for COP-MOP8, the CBD Secretariat reported some select information from these third national reports to suggest that the Guidance is being used or is useful. With regard to the answers to the third national reports aligned with the results of the testing of the Guidance, of the Parties that have conducted *any* risk assessments, 31 also participated in the “Testing” of the Guidance; Of those 31 Parties, 60% of these “agreed-4” or “strongly agreed-5” that the Guidance “is useful” in response to the testing, which might suggest that 60% of these Parties do consider the Guidance useful, as implied in the above referenced COP-MOP8 meeting document from the Secretariat. The average “agreement rating” among the 31 Parties was 3.4 that the Guidance is “useful or has utility.”

Box 6 **Third national report on implementation of the Cartagena Protocol on Biosafety.**

**Relevant questions on risk assessment**

Q. 85 Has your country adopted or used any guidance documents for the purpose of conducting risk assessment or risk management, or for evaluating risk assessment reports submitted by notifiers? a. Risk Assessment. b. Risk Management.
Q. 86 Is your country using the “Guidance on Risk Assessment of LMOs” (developed by the Online Forum and the AHTEG on Risk Assessment and Risk Management) for conducting risk assessment or risk management, or for evaluating risk assessment reports submitted by notifiers?
Q. 89 Has your country ever conducted a risk assessment of an LMO including any type of risk assessment of LMOs, e.g., for contained use, field trials, commercial purposes, direct use as food, feed, or for processing?
Q. 90 If you answered Yes to question 89, please indicate the scope of the risk assessments (select all that apply): Commercial Production; Field Trials; Contained Use; Food; Feed; Processing.

Upon a closer look at the third national reports, it can also be noted that, of the Parties that indicated having NOT conducted Risk Assessments, only ten also participated in the testing of the Guidance. Of those ten, all (100%) “agreed” or “strongly agreed” that the Guidance “is useful,” with an average agreement rating of 4.7, again demonstrating that Parties with less experience conducting risk assessments rated the Guidance higher than Parties with more experience. The third national reports also show that of those Parties that have conducted *any* type of Risk Assessment, 89% also reported having adopted or used *any* guidance documents for the purpose of conducting or evaluating risk assessments; and of those Parties that have adopted or used *any* guidance, 74% reported *not* using “the Guidance,” indicating that there is some other guidance that they are using.

Tables 3A,B show the answers to the questions on risk assessment from the third national reports against the testing scores on the Roadmap section of the Guidance. Table 3A includes the Parties that indicated they are conducting risk assessments when the scope of the assessment was for commercial production, and Table 3B includes those parties that have conducted risk assessments *not* for commercial production, or have not conducted any type of risk assessments. The testing scores for the Roadmap section of the Guidance, averaged across the four criteria, for all of the Parties and the non-Party governments who participated in the testing are shown in Tables 3A,B, along with the number of comments provided by each participant on the Roadmap section. Tables 3A,B also show the answers to the question from the third national reports asking whether the Party is using *any* guidance for risk assessment (Q85a), and whether the Party is using the Guidance developed by the AHTEG for conducting risk assessments (Q86) (see Box 6). In addition to the Parties shown in Tables 3A,B, 22 more Parties (not included in the tables because these did not participate in the testing) also reported in their third national reports using any guidance for risk assessment and not using the AHTEG Guidance^5^. The EU and all of the EU member states that submitted third national reports indicated that they are using guidance for risk assessment, and not using the AHTEG Guidance. This is predictable because the EU has a well-developed existing guidance for ERA of genetically modified plants (EFSA, 2010).

These trends from the third national reports against the results of the testing do seem to indicate that most Parties (developed or developing) that have conducted risk assessments are, in fact, *not* using the Guidance and agree less that the Guidance is “useful,” as for all other criteria for the testing, than Parties that have not conducted risk assessments. Most of these Parties that have conducted risk assessments have adopted and/or used other guidance documents for the purpose of conducting risk assessment rather than the Guidance developed by the AHTEG. Parties that have conducted risk assessments and have followed other guidance may have given lower scores in the testing because they have more experiences upon which to base their evaluation of the Guidance.

It is important to note these trends when considering the “usefulness” of the Guidance. While it stands to reason that Parties with limited experience in risk assessment are more in need of guidance, it also stands that Parties with more experience are in a better position to develop guidance based on that experience. Furthermore, many Parties “with experience” are in fact developing countries, and these developing countries with experience should not be conflated with Parties that are more in need of guidance. Perhaps more consideration of the experiences of Parties with actual cases of risk assessment and the other guidance documents these Parties have adopted and/or used, which seemed to be the original intent for the AHTEG, would have resulted in a more useful guidance document for the less experienced Parties, and one that could have been endorsed by the Parties.

### When Experts With Experience Test the Guidance

As it is, many of the “experts” participating in the development of the Guidance, whether from Parties or not, developing countries or not, on the AHTEG, in the online forum, and participating in the testing, although experts in their fields, had limited experience with “actual” risk assessments of LMOs upon which to base their contributions to these discussions. Recognizing this, as there was an open invitation after COP-MOP7 to “*Parties, other Governments, and relevant organizations to test or use, as appropriate, the Guidance in actual cases of risk assessment*” (Decision BSVII/12^1^), a workshop was organized and took place on Feb. 1-3, 2016 in Washington DC^6^, bringing together a group of individual experts who have worked with the regulatory authorities in countries that do have experience with “actual cases” of ERA for general release into the environment. The purpose of the workshop was to review the Roadmap from the Guidance at that stage and compare this information to actual cases of risk assessments in their country, noting how these fit into the steps outlined in Annex III of the Protocol (Box 1).

Individual experts who participated in the workshop were from the following countries: Argentina, Australia, Brazil, Canada, Colombia, European Union, India, Japan, Mexico, Netherlands, South Africa, and the United States. (Two participants from the US were from the two agencies involved in ERAs with two separate mandates: USDA APHIS and EPA). Individuals from these countries were selected to participate in this testing exercise based mainly on their personal experience as risk assessors in countries (from the list shown in Table 2) that had approved for commercial production more than one crop. Eight of the twelve participants had their experience from work in countries that are Parties to the Protocol, and four were from non-Parties (Argentina, Australia, Canada, US); also, five of the participants were from countries that are considered “developing countries” (Argentina, Brazil, Colombia, Mexico, South Africa)^2^.

Thus, the participants at this workshop were a good representation of countries with experience in conducting risk assessment, by individuals who had actual experience conducting risk assessments in their countries. None of the experts who participated were at that time members of the AHTEG. Some of the experts who were invited to participate had, during the online forums, expressed some concern about the Guidance as it was being developed, suggesting that what they observed in the Guidance did not align with their experiences with risk assessment. It should also be noted that all opinions shared during this workshop were understood to be that of the individuals based on their experience, and individuals were not asked or expected to represent the position of their government (Party or non-Party) in any way.

In order to conduct this “testing,” the experts were provided with a copy of the Roadmap (note, this was the version of the Guidance that was available as AnnexII in the report from the AHTEG (UNEP/CBD/BS/RARM/AHTEG/2015/1/4^1^) after its first meeting after COP-MOP7, in Brasilia, November 2017), and a template that captured all of the concepts in the Roadmap into a table, taken from the text of the Roadmap in the order they appeared there. The experts each chose a recent, actual case of risk assessment from their country to consider as they went through the concepts of the Roadmap to determine whether each concept is considered or not in the ERA that was actually done. This exercise served as a guide for each of the participants to present to the group how their risk assessments compare with the Roadmap. (Participants from Australia and Japan were not able to attend the workshop, but completed the evaluation and shared this for the discussion during the workshop).

#### The Outcome of the Testing by Experts With Experience

At certain points, it was difficult for the participants to say whether the concepts in the Roadmap were considered in their risk assessments. The participants noted that there are concepts in the Roadmap that may be considered during their risk assessment, but are not necessarily captured as part of the document that is finally produced from the ERA, and there were other concepts that were clearly only considered in certain cases. In a few cases, the participants struggled to understand the concept as it was described in the Roadmap. Yet, it is notable that nearly half of the concepts in the Roadmap are ones that most participants agreed are considered as part of their risk assessments, and there were only a few concepts (~10%) that most participants said they do *not* consider. The remaining concepts, however, were considered by some and not considered by others. This suggests that there is, among the different risk assessments in different countries, much in common, but also certainly much of the Roadmap that does not reflect a common approach among countries. Still, participants in this workshop agreed that many of the concepts captured in the Roadmap are indeed relevant to risk assessment.

Interestingly, the participants who thought that most (~90%) of the concepts from the Roadmap are considered in their risk assessments came from the EU, the Netherlands, South Africa, Japan, and the US. (In the case of the US, a concept was counted as “considered” if it was considered in risk assessments at either APHIS or EPA). The participants who thought that the least concepts are considered in their risk assessments were India and Argentina, although even these participants thought that more than half of the concepts are considered in their risk assessments. In the middle were Australia, Brazil, Canada, Colombia, and Mexico. There did not appear to be a clear separation between the participants whose experience was with Party governments and non-Party governments, or between the developed and developing countries with regards to the concepts in the Roadmap. This also suggests that experience in conducting risk assessment is more predictive of testers response to the Guidance than the overall economic development of their country or status as a Party. The results of this test also seem to suggest that it is not so much the concepts in the Guidance (or at least the Roadmap), but some other aspects that caused the concerns expressed by Parties at COP-MOP8.

The remainder of the workshop was devoted to discussion to elucidate this distinction, including some time working in smaller groups to consider possible changes to improve the various sections of the Roadmap. The overriding conclusion from these group discussions was that, although many of the concepts are included in their risk assessments, the roadmap simply does not reflect the “process” followed for risk assessment based on their experience. Ultimately, the participants were able to agree on a set of “consensus points” that summarize the major flaws in the Roadmap:

- Many basic concepts presented in the Roadmap are relevant for ERA, but the Roadmap is organized in a way that confounds the risk assessment process.
- The Roadmap does not capture the experience that has been gained in the last 25 years of LMO risk assessment.
- There is a lack of clarity on the objectives and the target audience for the Roadmap.
- The Roadmap is not always consistent with and goes beyond the scope of Annex III of the Cartagena Protocol.
- The level of detail and attention given to certain subjects in the Roadmap is disproportionate in terms of relevance to ERA.
- The treatments of uncertainty and monitoring in the Roadmap, in particular, are not consistent with Annex III, nor based on experience with risk assessment.
- The Roadmap is not appropriate for risk assessments related to field trials.
- Key terms and concepts should be more clearly identified, defined, and linked to existing terminology in general use for ERA, and put into the appropriate context.
- These concerns with the Roadmap should be adequately addressed and agreed upon before any additional guidance is considered.

Most of these same points are also reflected among the comments submitted with the results of the testing and in the online forum discussions. In general the participants of the workshop did not see an easy way to address these flaws through straight-forward revisions or rearrangements in the text. Therefore, the result of this testing led to a conclusion that the Roadmap is not practical or useful as a guide for risk assessment and the problems with it cannot be easily fixed. Although this workshop took place before the final revisions by the AHTEG were presented at COP-MOP8, the problems identified by this expert group remained in the final version.

## Conclusions

The AHTEG completed its mandate to work on the Guidance on Risk Assessment of LMOs by COP-MOP8 in 2016, where the COP-MOP “took note of” the Guidance, but did not endorse it, calling it “voluntary” Guidance in the decision, making it available but entirely clear that there is no obligation by Parties to use this Guidance. Although the work on the Guidance in its current form is finished, the work on risk assessment under the Cartagena Protocol continues. There was a decision at COP-MOP8 (Decision BSVIII/12^1^) to extend the online forum on Risk Assessment and Risk Management to continue to exchange experiences on risk assessment, provide information and views on perceived gaps in existing guidance materials, and provide proposals to address any gaps identified. In the discussions that ensued, some Parties submitted requests for additional guidance on specific issues while other Parties made a case that no additional guidance is needed at this time, a difference of opinion that had been expressed continuously throughout the process to develop the Guidance.

Therefore, the decision for further work on risk assessment coming out of the most recent COP-MOP9 (CBD/CP/MOP/9/13) which took place in Sharm-el Sheik, Egypt in November of 2018, focuses on developing a “process” to identify and prioritize the specific issues, if any, of risk assessment for consideration by COP-MOP10 before there will be any decision to develop any further guidance. In effect, the request from COP-MOP9 is responding to the fact that, in addition to the development of a dysfunctional Roadmap, development of further guidance on additional topics had already been attempted by the AHTEG on a rather arbitrary list of specific issues, i.e., on stacked genes, abiotic stress, mosquitos, trees, and monitoring, and proposed for fish and synthetic biology, without a clear process in place for selecting these issues. A clear process and criteria for identifying and prioritizing specific issues for developing further guidance on risk assessment is absolutely essential, and one critically important criteria to consider, as described in Annex I of the COP-MOP9 Decision, is whether a topic or issue poses challenges to existing risk assessment frameworks, guidance, and methodologies. Had existing risk assessment approaches been given due consideration before the Guidance was pursued initially, there may have been considerable savings in time, energy and money.

However, if it is decided that further guidance on a specific issue is needed, a more important decision by the COP-MOP will be about the proper process for developing that guidance and how to include the most relevant and appropriate expertise. The COP-MOP must consider whether an AHTEG functioning according to the consolidated modus operandi of the SBSTTA and rules of procedure of the Convention, as was this past AHTEG, is the most appropriate body of experts to develop such guidance. Clearly, the outcome from the work of the AHTEG and online forum on Risk Assessment and Risk Management tells us that something must be changed in this process. At a minimum, the COP-MOP must develop a means of separating political discussions from an undertaking by technical experts. There must be a more effective way a group of experts can develop guidance that represents consensus on a technically sound approach to risk assessment, or a way to capture in the outcome the differences of opinion that might be more meaningful as guidance to Parties, rather than delivering a compromise document.

The experience with the Guidance on Risk Assessment of LMOs, as described herein, seems to indicate that the only way to reach agreement among Parties is not to base any further guidance on what experts think “should be done,” but to base it on commonalities from experiences with existing, actual cases of risk assessment. In this case, risk assessment guidance under the Cartagena Protocol could only be developed on specific issues where there is experience with risk assessment and when Parties can agree that the guidance being developed represents their experience. Many Parties have the opinion that many of the specific topics that have been identified to date could be assessed for risk based on an extension of current practices. This includes some applications of synthetic biology, genome-editing, and gene drive systems in living modified organisms, all currently topics of discussion for risk assessment under the Convention and its Protocol. With respect to risk assessment, the many possible applications of these technologies must be considered on a case-by-case basis.

In fact, it is currently and should remain the responsibility of Parties, within their own domestic frameworks, to determine how to do risk assessment that is consistent with Annex III of the Protocol and national environmental policy. Ultimately, Parties with less experience may do better to identify and choose Parties with more experience from which they may learn, in order to develop guidance on risk assessment that meets their specific needs while remaining consistent with their obligations under the Protocol. The COP-MOP may do better to devise ways to support this sort of Party-to-Party assistance, or to invite other less constrained input from experts with experience, rather than putting limited resources into a process that may be correct according to policy, but in practice is destined to fail.

## Author Contributions

KH collected and analyzed the information and wrote the manuscript based on the documents referred to within, and on personal observation as an observer on the AHTEG for Risk Assessment and Risk Management from 2012 to 2016 and at COP-MOPs 2-9.

### Conflict of Interest Statement

The author declares that the research was conducted in the absence of any commercial or financial relationships that could be construed as a potential conflict of interest.

## Footnotes

^1^All of the COP-MOP meeting documents referenced throughout this manuscript and other relevant information can be found online through the Biosafety Clearing House of the Cartagena Protocol on Biosafety. https://bch.cbd.int/protocol/.

^2^All countries identified as ‘developing countries’ throughout this paper are listed as ‘developing economies’ or ‘economies in transition’ according to the World Economic Situation and Prospects of the United Nations (United Nations, 2018). This is presumably the list used by the CBD secretariat to identify ‘developing countries’ in its analyses and reports. More than three quarters of the Parties to the Protocol could be considered developing countries according to this list.

^3^Third national reports on implementation of the Protocol were submitted by 128 Parties to the Executive Secretary of the CBD before MOP8 (in December of 2016). Copies of all third national reports as submitted are available on the Biosafety Clearing House^1^.

^4^The Protocol uses the terminology ‘intentional introduction into the environment’, but does not clearly distinguish introduction for confined field trials from introduction for commercial production (which was incidentally one consistent point of disagreement related to the purpose and scope of the Guidance).

^5^Burkina Faso (D), Bulgaria (D), Cameroon (D), Estonia (EU), Finland (EU), France (EU), Ghana (D), Guatemala (D), Indonesia (D), Lithuania (EU), Malawi (D), Nicaragua (D), Romania (EU), Slovakia (EU), South Korea, Sweden (EU), Switzerland, Tanzania (D), Thailand (D), Uganda (D), United Kingdom (EU), Zimbabwe (D).

^6^The workshop was organized by the University of Minnesota Stakman-Borlaug Center for Sustainable Plant Health and was supported primarily through a grant from the USDA National Institute of Food and Agriculture, Biotechnology Risk Assessment Grant Program (Grant no. 2015-33522-24097 awarded to PI K. Hokanson, University of Minnesota).
